# Science capital: Results from a Finnish population survey

**DOI:** 10.1177/09636625241310756

**Published:** 2025-02-14

**Authors:** Johanna K. Kaakinen, Sari Havu-Nuutinen, Tuomo Häikiö, Hanna Julku, Teija Koskela, Mirjamaija Mikkilä-Erdmann, Milla Pihlajamäki, Daria Pritup, Kirsi Pulkkinen, Katri Saarikivi, Jaana Simola, Valtteri Wikström

**Affiliations:** University of Turku, Finland; University of Eastern Finland, Finland; University of Turku, Finland; University of Helsinki, Finland; University of Turku, Finland; University of Helsinki, Finland; University of Turku, Finland; Metropolia University of Applied Sciences, Finland; University of Turku, Finland; University of Helsinki, Finland; University of Turku, Finland

**Keywords:** adult education, science attitudes, science capital, science teaching, science self-efficacy

## Abstract

This study examined science capital among Finnish adults (*N* = 1572), who responded to 37 survey items assessing science capital. Factor analysis suggested four science capital dimensions: visiting science-related places, science attitudes, science-related self-efficacy, and early support for studying natural sciences. Higher education and higher parental education were linked to higher science capital across all dimensions. Older participants exhibited lower science-related self-efficacy, less early support, and more negative science attitudes than younger respondents. Age and education were stronger predictors of science-related self-efficacy and early encouragement for men than women, and mothers’ education had a weaker effect on science-related self-efficacy for men. The results show that science capital is a multidimensional construct and highlights that younger generations in Finland have had more opportunities to develop their science capital. These findings emphasize the need for early and equitable support to foster positive science attitudes and participation.

Societies are to a growing degree confronted with significant and complex global phenomena such as pandemics and the consequences of climate change, which call for citizens’ better understanding of scientific information and its use in everyday decision-making (Sinatra and Hofer, 2016, 2021). For example, mitigating the consequences of climate change requires that citizens understand and accept the scientific evidence showing that climate change is real, that it is caused by human activities, and that its consequences are potentially catastrophic to the Earth’s ecosystems and our civilization. However, there is a gap between existing scientific knowledge and public acceptance of scientific facts (Capstick et al., 2015). Moreover, it seems that many people do not see the relevance of scientific knowledge: on average, 32% of European citizens think that knowing about science is not important in their daily lives (Eurobarometer, 2021).

Knowing more about the factors that impact interest and attitudes toward science could help bridge the gap between scientific knowledge and the public’s perceptions and everyday actions (Sinatra and Hofer, 2016, 2021). Previous large-scale international surveys of public opinion and understanding of science (e.g. Eurobarometer, 2021; Pew Research Center, 2015) provide a general picture of how science and scientists are perceived, and what people know about science (see Besley, 2013). In the present study, we adopt the perspective of *science capital* (Archer et al., 2015), which may also help to understand the reasons for low acceptance of scientific facts and negative attitudes toward science. By employing the science capital lens, we especially aim to reveal how people experience, interact with, and participate in science and scientific activities in their everyday lives (Archer et al., 2015). This knowledge will inform the design of interventions and policies that aim to increase the acceptance and use of scientific information in society.

## 1. What is science capital?

Science capital extends the Bourdieusian notions of *social* and *cultural capital* (Bourdieu, 1984) to science (Archer et al., 2015, 2014). According to Archer et al. (2015), science capital consists of *science-related cultural and social capital* and *science-related behaviors and practices*. Science-related cultural capital includes science literacy, which refers to an individual’s scientific knowledge and skills, ability to make use of scientific knowledge, and understanding of the scientific method, or “how science works” (Archer et al., 2015). In addition to scientific knowledge, science-related cultural capital also includes science-related dispositions and preferences, including positive attitudes toward science and appreciation of scientific information. These start to develop already in childhood, and they are influenced by the family environment and experiences with teachers in formal education (Archer et al., 2015). Finally, science-related cultural capital contains symbolic knowledge about the transferability of science in the labor market, which refers to seeing science qualifications as beneficial for one’s career (Archer et al., 2015).

Science-related social capital consists of various forms of social resources and behaviors. These include things like knowing someone who works in a scientific occupation, parental science qualifications, and preference to talk to others about science (Archer et al., 2015). Science-related social capital is closely intertwined with science-related cultural capital. For example, a family’s socio-economic group and social networks may impact its children’s appreciation for science and their aspirations as well as opportunities to seek science education and pursue a scientific career (see e.g. Armila et al., 2018).

Finally, in addition to science-related social and cultural capital, Archer et al. (2015) identified science-related behaviors and practices as an important aspect of science capital. These include the consumption of science-related media and participation in informal science learning.

According to Archer and colleagues (2015, 2014), science capital is produced and reproduced by the different dimensions of cultural and social capital. This means, for example, that individuals who possess high levels of science-related social capital, such as being members of social networks that value science, are more likely to also gain science-related cultural capital like having a positive view of science and its utility, and to engage in science-related activities that build up science knowledge. Such a self-perpetuating cycle will eventually lead to greater differences among people and, potentially, to polarization in who can access and benefit from scientific information. Dominant socio-economic groups have privileged access to cultural and social capital (Claussen and Osborne, 2013), and thereby more possibilities to develop science capital. However, research has shown that science capital is distinct from cultural or social capital because it can be acquired regardless of one’s social, economic, or cultural status—meaning that it is possible to acquire high science capital despite low cultural capital (e.g. Archer et al., 2015; Moote et al., 2021).

As science capital is a broad concept that covers science-related attitudes, dispositions, experiences, and behaviors, it has the potential to offer new insights into why some people can make use of and benefit from science and scientific information more than others. Examining the components of science capital and their interrelations will increase awareness of science capital and provide valuable information for developing interventions that foster science equity.

But how to measure such a complex concept as science capital? Previous empirical research has aimed at developing instruments for measuring science capital among adolescents and youth (Archer et al., 2015; Jones et al., 2021). Based on the Bourdieusian theory, Archer et al. (2015) devised a set of survey questions that tapped into science-related cultural and social capital and science-related behaviors. Archer et al. (2015) identified nine components that captured the variance in the survey questions: (1) future science aspirations, (2) valuing science and scientists, (3) family attitudes and expectations, (4) utility of science qualifications, (5) participation in informal science activities, (6) science media engagement, (7) valuing museums and museum experiences, (8) experiences with science teachers and lessons, and (9) self-efficacy in science. They then identified individual survey questions that best predicted the main outcome variables reflecting high or low science capital: future science career aspirations and whether other people recognize the respondent as a “science person.” Based on the 14 items that best differentiated high and low science capital respondents as defined by their responses to the outcome variables, they computed a *science capital index*, a single score that reflects an individual’s science capital (see also DeWitt et al., 2016).

Recently, Jones et al. (2021) developed the *NextGen Scientist Survey* for measuring science capital in youth to understand factors underlying individual differences in career aspirations. The survey combined questions used in different large-scale surveys (Archer and DeWitt, 2013; Fraser, 1981; Schreiner and Sjøberg, 2004; Unfried et al., 2015), and its reliability and validity were examined in a sample of middle school students. The data suggested that science capital consists of four correlated dimensions: (1) expectancy value, (2) science experiences, (3) future science task value, and (4) family science achievement value. These four dimensions corresponded to the components found in the study by Archer et al. (2015; see also DeWitt et al., 2016). The items in the expectancy value factor relate to science self-efficacy and identity, supporting Archer et al.’s (2015; DeWitt et al., 2016) view that one’s perceptions of self in relation to science and whether other people see them as a “science person” form an important aspect of science capital. Science experience items tapped into voluntary and informal modes of science activities, including science media engagement, aligning well with the idea that science engagement outside of school contributes to science capital (Archer et al., 2015; DeWitt et al., 2016). Future science task value questions corresponded to Archer et al.’s (2015; DeWitt et al., 2016) questions on future career aspirations, and whether one thinks they will need science in their future job. Finally, family science achievement value items scoped family attitudes and expectations, which have been identified as important in shaping science capital in other studies (Archer et al., 2015; DeWitt et al., 2016).

These findings (Archer et al., 2015; DeWitt et al., 2016; Jones et al., 2021) show that in youth, science capital is composed of a complex mixture of attitudes and perceptions of science and its utility (Mujtaba et al., 2018), self-efficacy in science (Turnbull et al., 2020), support and expectations of family members (Archer et al., 2012; Mujtaba et al., 2018), how teachers and school lessons inspire and encourage students to engage in science (Archer et al., 2020, 2017; Turnbull et al., 2020), and how much informal science activities students can or want to participate in (DeWitt and Archer, 2017; Mujtaba et al., 2018).

However, while the studies identify similar aspects underlying science capital in youth, the major difference between them is that some studies conceptualize science capital as a unitary construct (Archer et al., 2015), whereas others show that it is composed of separate intercorrelated dimensions (Jones et al., 2021). This difference is theoretically important and has consequences for how science capital should be measured and investigated. The unidimensional view assumes that a single score suffices because the underlying dimensions are so tightly associated that certain factors, like family’s socio-economic group, should have similar effects on all of them. The multidimensional view posits that the different dimensions of science capital should be measured separately, and that different factors may influence these dimensions. For example, family’s socio-economic group might be strongly associated with one dimension but not with another.

Previous research on science capital has largely aimed to improve understanding of how formal education shapes the science career aspirations of adolescents and youth (e.g. Archer et al., 2015, 2014; DeWitt et al., 2016; Jones et al., 2021), whereas very little is known about what constitutes science capital in adulthood and how it is accumulated outside formal education (see Kontkanen et al., 2023). Science capital is not fixed in childhood or adolescence: one can gain or lose capital, as it is shaped by the social and cultural context and through education (Aschbacher et al., 2010; Moote et al., 2021). Understanding dimensions that underlie science capital in adulthood is of crucial importance when crafting actions and policies that increase the acceptance and use of scientific information in society. In the present study, we aimed to fill this gap in the research field and study science capital and its distribution in the adult population. To do that, we developed the *Science Capital Questionnaire* (SCQ), and examined how socio-demographic factors are associated with science capital.

## 2. Distribution of science capital

Systematic research on the distribution of science capital in adults is scarce, but previous research on children and youth shows that socio-demographic factors play an important role in who accumulates science capital. For example, boys have higher science capital than girls (e.g. Archer et al., 2015), and the gender gap is likely carried over to adulthood (Moote et al., 2021). The results of a recent large-scale survey (Eurobarometer, 2021) show that even though men and women are equally interested in learning more about scientific discoveries, more women (50%) than men (42%) say that science is so complicated that they do not understand much about it, indicating that women may have lower science self-efficacy. Interestingly, the gender gap in science capital persists when comparing men and women who are studying in science, technology, engineering, and mathematics (STEM) fields, as female STEM students have lower self-efficacy in science than male students (Kelly et al., 2019; Turnbull et al., 2020). This means that despite being highly educated, even in a science field, women may still not perceive themselves as “science persons.”

Education is very likely a crucial factor in acquiring science capital. Highly educated citizens are more likely to have positive attitudes and dispositions toward science (e.g. Eurobarometer, 2021; Noy and O’Brien, 2019), have higher science self-efficacy (e.g. Gutwill, 2018), and participate in voluntary science-related activities (e.g. Edwards et al., 2018; Wilson-Lopez et al., 2018), which all are expected to contribute to science capital. As family background is assumed to have a strong impact on the accumulation of science capital across the life span, parents’ education level may be reflected in science capital in adulthood. Children of educated families are more likely to get into higher education and be steered toward a science career (e.g. Smith and White, 2011). They also have more positive attitudes and dispositions toward science (Archer et al., 2012), have higher science self-efficacy (Turnbull et al., 2020), and are more likely to attend voluntary and informal science activities (DeWitt and Archer, 2017). However, some previous research suggests that the influence of family background on science career aspirations of youth is indirect and mediated by science self-efficacy (Halim et al., 2021). Moreover, research suggests that parental attitudes toward science do not predict science self-efficacy in university students (Turnbull et al., 2020), suggesting that the role of family attitudes may diminish in adulthood.

An interesting question is whether science capital differs between adult age groups. Results of a survey conducted among 17–18-year-olds suggest that, in comparison to younger age groups, the number of students with low science capital is higher (Moote et al., 2021). Science capital thus seems to decrease with age. Large-scale surveys of science attitudes among adults suggest that younger and older adults have different views on many scientific topics, such as human impact on climate change (Pew Research Center, 2015). Younger adults are more interested in new scientific discoveries and technological developments than older adults, but older adults show a higher interest in new medical discoveries (Eurobarometer, 2021). However, older adults are more likely than younger adults to think that science is so complicated that they do not understand much about it and that science is not important in their daily lives (Eurobarometer, 2021). These findings indicate that older adults are more likely to have lower science self-efficacy and more negative dispositions toward science than younger adults.

## 3. Aims and research questions

This study aims to expand the understanding of the validity and multidimensionality of the empirical concept of science capital, gain insights into how social and cultural science capital accumulates, and provide suggestions for actions and policies that increase the acceptance and use of scientific information in society. Our study was set to answer two research questions: (1) what are the dimensions of science capital in adulthood, and (2) how do gender, age, education, and parental education relate to science capital?

We examined these questions in Finland, a northern European democratic welfare state with a population of approximately 5.6 million. Finland offers a unique context for examination and comparison of the factors affecting dispositions toward science. Education in Finland is free at all levels, and there is high trust in the public educational system (see Välimaa, 2021). Finnish youth have been consistently scoring above the Organisation for Economic Co-operation and Development (OECD) average in science in large-scale global skill assessments such as Programme for International Student Assessment (PISA), even though these have shown a declining trend recently (OECD, 2023). Previous large-scale surveys on attitudes and dispositions toward science consistently show that Finns have relatively positive perceptions of science and scientists (Eurobarometer, 2021). We can thus examine whether the data collected in a Nordic welfare state with a robust public educational system show previously detected patterns of, for example, unequal distribution of science capital. As education is free, it allows uncovering how parental and individuals’ own education influences accumulation of science capital beyond access to formal education due to economic circumstances.

## 4. Methods

### Participants

Participants were recruited using random sampling of 18–75-year-old people living in mainland Finland. A random sample of 8500 Finnish residents was obtained from the Population Information System provided by the Digital and Population Data Services Agency, and the survey was mailed to their home address. The sample was representative of the Finnish population considering age, gender, and geographical location of the home address. Out of the 8500 persons who were contacted, a total of 1572 participants (18.49%) responded to the survey by the end of the data collection period (end of October 2021), either by mailing the questionnaire to the return address (73.09% of the respondents) or by completing it online (26.91%). The demographic data used in the analyses are based on the respondents’ self-reported background information (see Table 1). The final sample was mostly representative of the Finnish population (see the Supplemental Material), except for females being overrepresented. However, to maintain the interpretability of the results, we did not apply weighting to correct for the gender bias in the sample.

**Table 1.** Descriptive statistics of the demographic variables.

Variable	*M*	*SD*	
Age (years)	51.31	16.94	
Categorical variable	Category	*N*	%
Gender	Female	808	51.40
	Male	702	44.66
	Other	4	0.25
	Missing	58	3.69
Home language	Finnish	1361	86.58
	Swedish	68	4.33
	English	25	1.59
	Russian	19	1.21
	Other	31	1.97
	Missing	68	4.33
Education	Basic/primary	132	8.40
	Secondary school	143	9.10
	Vocational degree	384	24.43
	Bachelor’s degree	374	23.79
	Master’s degree	284	18.07
	Licentiate or PhD	46	2.93
	Other	83	5.28
	Missing	126	8.02

### Science Capital Questionnaire

The survey questions were based on previous science capital items developed in the longitudinal ASPIRES project (J. DeWitt, personal communication, 2021; University College London, 2022) and science capital surveys conducted in the United Kingdom (Archer et al., 2015; UK Department for Business, Energy & Industrial Strategy, 2019). The questionnaire included 37 items tapping into different aspects of science-related social and cultural capital as well as science-related activities, as identified in previous research (Archer et al., 2015; Motta et al., 2021; UK Department for Business, Energy & Industrial Strategy, 2019; University College London, 2022). The items and how they correspond with dimensions of science capital identified in previous studies are described in Table A in the Supplemental Material.

As some of the questions were originally designed to be used with adolescents, we modified certain sections to suit adult respondents by adopting a retrospective view: respondents were instructed to think back to their childhood or school years (e.g. “Think of your time in school”). Furthermore, as the English term *science* is strongly associated with STEM subjects, while the Finnish term *tiede* (science) also includes all disciplines including humanities and social sciences, we adopted this all-inclusive concept of science throughout most of the survey and adjusted the questions accordingly. However, to preserve the original wording of items as much as possible, items concerning support in STEM subjects during school years were specified as relating to “natural sciences” taught in Finnish schools, that is physics, chemistry, biology, and geography.

The questions were answered on a 5-point Likert-type scale (1 = completely disagree, 2 = somewhat disagree, 3 = neither disagree nor agree, 4 = somewhat agree, 5 = completely agree) apart from two questions. The question about visiting science-related places or participating in science-related activities was answered on a 6-point scale (1 = not at all, 2 = sometimes, 3 = once per year, 4 = twice per year, 5 = three to four times per year, 6 = five times per year or more). As the data were collected during the COVID-19 pandemic, this question was modified to reflect the time before the pandemic. The question about knowing people in science had three response options: (1) yes, (2) no, and (3) I don’t know.

The questions were translated into Finnish and back-translated into English until the research group agreed the questions resembled their original counterparts as closely as possible. Members of the research group with high proficiency in Swedish, English, and Russian translated the final questionnaire into these languages. Translations into indigenous (Northern Sami) and other languages most frequently spoken in Finland (Estonian, Arabic, and Somali) were done by professional translators. The English version of the questionnaire is available in the Open Science Framework (https://osf.io/dkwqr/).

The questions included in the survey were piloted in April 2021 with 17 Finnish-speaking participants, who were interviewed via phone or Internet call (40–60 minutes per interview). During the interviews, participants were asked to evaluate whether the questions were understandable and interpretable in the way the research group intended. Questions that were difficult for respondents to understand, for instance, because of specialized or ambiguous terminology, were revised based on the interviews.

### Background information and other survey questions

The background information included questions about gender, age, home language, perceived minority group status, education, occupation, hobbies, and annual household income.

In addition to the SCQ, the survey included other scales on, for example, thinking styles, active participation in society, and the use of media. However, these scales will not be used in the present analyses and thus, they are not described in more detail here.

### Procedure

The study was approved by the Ethics Committee for Human Sciences of the University of Turku and followed the Declaration of Helsinki. The first batch of questionnaires was mailed in May 2021. Reminder letters to those who had not answered by the end of July 2021 were mailed in August 2021. Participants received the materials in their native language if the Population Information System data indicated it to be one of the following: Finnish, Swedish, English, Northern Sami, Russian, Estonian, Arabic, or Somali. If their native language was indicated to be something else, participants received the materials in English. Participants were informed that they could respond to the survey either on paper or via the Internet. Four gift certificates (50€) to local retail stores were drawn among all participants who participated in the draw by the end of October 2021.

### Statistical analyses

The factor structure underlying the SCQ was examined in two steps (see Schmitt et al., 2018). We first evaluated the appropriate factor structure with exploratory factor analyses (EFAs) on a randomly selected 50% of the data. Next, we tested the factor structure suggested by the EFA with a confirmatory factor analysis (CFA), which was applied to the remaining 50% of the data. CFA was then used to examine whether the survey items reflect a unidimensional or a multidimensional construct by comparing a model with a superordinate latent construct with a model including correlated first-order dimensions.

After we had established an acceptable measurement model for the SCQ, we examined the associations between socio-demographic variables and SCQ in the whole data. We first conducted measurement invariance tests, which showed that we did not obtain metric invariance between gender groups. Thus, we conducted groupwise structural equation models (SEMs) to examine how age, education, and parents’ education predict science capital for men and women.

More detailed information about the statistical analyses and handling of missing data is provided in the Supplemental Material. Data and analysis code are available at https://osf.io/dkwqr/.

## 5. Results

### What are the dimensions of science capital in adulthood?

Descriptive statistics for the items included in the SCQ in the final sample are presented in Table 2. After examining the different factor solutions suggested by EFAs and CFA model comparisons between a model with four first-order factors and a model with a superordinate factor (see the Supplemental Material), the final model consisted of four first-order factors (see Table 3). The model was a good fit to the data (Hu and Bentler, 1999): comparative fit index = .980, Tucker–Lewis index = .978, root mean square error approximation (RMSEA) = .068, 90% confidence interval for RMSEA = [.066, .071], standardized root mean square residual = .062. Correlations between the factors varied between |*r*| = .391 (Factors 1 and 3) and |*r*| = .859 (Factors 2 and 4).

**Table 2.** Descriptive statistics for the items included in the Science Capital Questionnaire.

Item	*N*	*M*	*SD*	Min	Max
Do you have relatives, friends, or colleagues, who work with/in science?^a^	1504	.47	.50	0	1
I speak about things related to science with other people.	1440	3.59	1.44	1	6
I’m well informed about science, scientific research and their developments.	1502	3.19	1.09	1	5
I’m interested in scientific research and findings.	1506	3.90	1.02	1	5
I follow news about new technologies.	1499	3.59	1.10	1	5
Science is not for me.	1488	1.99	1.12	1	5
The next generation has more opportunities for work due to science and technology.	1493	3.94	0.94	1	5
The scientific knowledge I learned in school has been useful in my daily life.	1497	3.51	1.13	1	5
The mathematics I learned in school has been useful in my daily life.	1501	3.78	1.07	1	5
Young people’s interest in science is essential for our future welfare.	1500	4.31	0.76	1	5
I don’t think I’m smart enough to understand science.	1504	2.24	1.12	1	5
I don’t think I’m smart enough to understand technology.	1502	2.28	1.15	1	5
Science has so much significance in our lives that everyone should be interested in it.	1500	3.73	1.00	1	5
School put me off science.	1498	2.05	1.06	1	5
Scientific knowledge is useful in my daily life.	1500	3.84	0.97	1	5
I don’t actually know what a scientist does.	1497	2.04	1.04	1	5
I don’t actually know what an engineer does.	1500	1.88	1.04	1	5
I would feel comfortable in places where science is discussed and practiced, such as laboratories, science centers, and industrial environments.	1500	3.33	1.16	1	5
I can well understand scientific terminology, such as hypothesis, theory, experiment, and clinical trial.	1505	3.71	1.16	1	5
Science is important for understanding the world.	1509	4.22	0.84	1	5
It’s important for our society that young people understand science.	1508	4.36	0.66	1	5
My parents or guardians thought it was important for me to study science in school.	1509	3.18	1.20	1	5
My parents or guardians were interested in science.	1506	3.05	1.19	1	5
My parents or guardians emphasized that science would be beneficial for me in the future.	1507	2.91	1.22	1	5
My teachers emphasized that science would be beneficial for me in the future.	1506	3.33	1.12	1	5
A significant adult (e.g. parent or teacher) encouraged me to continue science studies.	1507	2.55	1.28	1	5
My teachers thought I was gifted in science.	1506	2.81	1.22	1	5
How often do you usually visit the following places or participate in the following activities in your free time?					
Places related to science (e.g. observatories or botanical gardens)	1491	2.33	1.10	1	6
Science or technology museums	1495	2.02	0.88	1	6
Art museums	1487	2.70	1.46	1	6
Other museums (e.g. local or arts and crafts museums)	1494	2.59	1.20	1	6
Science centers	1498	1.87	0.74	1	6
Planetariums	1492	1.73	0.63	1	6
Zoos or aquariums	1501	2.19	0.83	1	6
Lectures, talks, or webinars related to science or technology	1495	1.82	1.19	1	6
Literature events	1486	1.67	0.99	1	6
National parks or other nature sites	1487	3.61	1.61	1	6

a This variable is categorical, in which 0 = no or I don’t know and 1 = yes.

**Table 3.** CFA model estimates of the four-factor model with full data.

Factor	Item	Estimate	*SE*	*z*
F1: Science-related activities	I visit places related to science (e.g. observatories or botanical gardens) in my free time.	1.00		
	Art museums	0.81	0.02	53.27
	Other museums (e.g. local or arts and crafts museums)	0.71	0.01	47.67
	Science or technology museums	1.05	0.02	63.57
	Science centers	0.97	0.02	62.32
	Literature events	0.69	0.02	44.01
	Planetariums	0.71	0.01	48.98
	Lectures, talks, or webinars related to science	0.91	0.02	56.17
	National parks or other nature sites	0.69	0.02	46.26
Factor 2: Negative self-efficacy	I don’t think I’m smart enough to understand science.	1.00		
	I don’t think I’m smart enough to understand technology.	0.92	0.02	58.33
	I don’t actually know what a scientist does.	0.96	0.02	57.90
	I don’t actually know what an engineer does.	0.73	0.02	45.14
	Science is not for me.	1.13	0.02	64.30
	I can well understand scientific terminology, such as hypothesis, theory, experiment, and clinical trial.	–1.09	0.02	–63.29
	I’m well informed about science, scientific research, and their developments.	–1.14	0.02	–65.78
	School put me off science.	0.80	0.02	50.88
	I would feel comfortable in places where science is discussed and practiced, such as laboratories, science centers, and industrial environments.	–1.04	0.02	–62.30
	I have relatives, friends, or colleagues, who work with/in science.	–0.91	0.02	–48.66
	I speak about things related to science with other people.	–1.06	0.02	–62.79
	I follow news about new technologies.	–0.94	0.02	–58.20
Factor 3: Early encouragement	My parents or guardians emphasized that science would be beneficial for me in the future.	1.00		
	My parents or guardians thought it was important for me to study science in school.	0.98	0.01	70.11
	A significant adult (such as parent or teacher) encouraged me to continue science studies.	0.91	0.01	68.47
	My parents or guardians were interested in science.	0.88	0.01	70.38
	My teachers emphasized that science would be beneficial for me in the future.	0.82	0.01	63.99
	My teachers thought I was gifted in science.	0.83	0.01	64.31
Factor 4: Science attitudes	Young people’s interest in science is essential for our future welfare.	1.00		
	It’s important for our society that young people understand science.	1.05	0.02	50.02
	Science has so much significance in our lives that everyone should be interested in it.	0.86	0.02	45.95
	Science is important for understanding the world.	1.26	0.02	56.15
	Scientific knowledge is useful in my daily life.	1.21	0.02	56.22
	The scientific knowledge I learned in school has been useful in my daily life.	1.13	0.02	54.49
	The next generation has more opportunities for work due to science and technology.	1.38	0.02	59.26
	I’m interested in scientific research and findings.	0.69	0.02	38.93
	The mathematics I learned in school has been useful in my daily life.	1.05	0.02	50.02
		Factor covariances		
Factor 1	Factor 2	–0.33	0.01	–54.05
	Factor 3	0.29	0.01	44.21
	Factor 4	0.27	0.01	44.99
Factor 2	Factor 3	–0.32	0.01	–54.12
	Factor 4	–0.37	0.01	–52.86
Factor 3	Factor 4	0.29	0.01	46.38

For all estimates, *p* <.05.

Items that loaded on Factor 1 were related to the frequency of visiting science-related places, and we labeled it as “Science-related activities.” Note that in addition to visiting places related to natural sciences, Factor 1 included visits to art museums and literature events, reflecting our wider conceptualization of science as also including humanities. Factor 2 included items related to negative science self-efficacy (e.g. “I don’t think I’m smart enough to understand science”), and we labeled it as “Science-related self-efficacy.” This factor also included items that have previously been labeled as science literacy items, such as “I can well understand scientific terminology, such as hypothesis, theory, experiment, and clinical trial.” Factor 3 included items related to support from teachers and parents when the respondent was at school, and we termed it as “Early encouragement.” Factor 4 consisted of items on science attitudes and dispositions (e.g. “It is essential for our future that young people are interested in science”), and we coined it as “Science attitudes.”

The pattern of correlations between latent factors shows how different dimensions are intertwined. The high correlation (*r* = –.859) between science-related self-efficacy and early encouragement indicates that early experiences at home and school are important in shaping self-efficacy and identity in adulthood. On the other hand, the weak correlation (*r* = .391) between science-related activities and science attitudes indicates that a positive disposition toward science is not strongly linked to participation in science-related activities. This finding highlights that science capital is not a uniform construct and that its different dimensions should be considered separately. The other correlations were moderate (|*r*|s = .517–.568), showing that science-related activities, self-efficacy, attitudes, and early encouragement are separate, yet correlated constructs.

### How do gender, age, education, and parental education relate to science capital?

We next conducted SEMs to examine whether background variables of age, education level, and parental education level predict the different dimensions of science capital. As we failed to establish measurement invariance between gender groups (see the Supplemental Material), these analyses were conducted separately for women and men. Moreover, parents’ education levels were highly correlated (χ^2^(16) = 1085.6, *p* < .001), and to avoid problems related to multicollinearity, the effects of the mother’s and father’s education were tested in separate models.

The regression slopes for the SEM model with age, education, and mother’s education as predictors of science capital dimensions, separately for women and men, are presented in Table 4. Age was not correlated with participating in science-related activities, but it had a positive association with negative science-related self-efficacy, and negative association with early encouragement and science attitudes, indicating that older respondents had more negative science-related self-efficacy, reported having experienced less early encouragement, and had more negative science attitudes than younger respondents. The comparisons of the regression slopes for men and women indicated that age had a stronger association with negative science self-efficacy (χ^2^(1) = 48.93, *p* < .001) and experienced early encouragement (χ^2^(1) = 22.35, *p* < .001) for men than for women.

**Table 4.** Regression slopes of the SEM predicting latent factors of science capital with age, education level, and mother’s education level with gender as a grouping factor.

Factor	Predictor	Gender group					
		Women			Men		
		Estimate	*SE*	*z*	Estimate	*SE*	*z*
F1: Science-related activities	Age	0.00	0.00	1.95	–0.00	0.00	–1.55
	Education	–0.22	0.02	–14.91*	–0.26	0.02	–14.21*
	Mother’s education	–0.06	0.02	–3.51*	–0.02	0.02	–0.81
F2: Negative self-efficacy	Age	0.00	0.00	4.57*	0.01	0.00	12.71*
	Education	0.19	0.01	19.45*	0.23	0.01	19.76*
	Mother’s education	0.05	0.01	4.74*	–0.00	0.01	–0.04
F3: Early encouragement	Age	–0.00	0.00	–3.33*	–0.01	0.00	–9.25*
	Education	–0.11	0.01	–7.79*	–0.17	0.02	–10.74*
	Mother’s education	–0.04	0.02	–2.66*	–0.03	0.02	–1.73
F4: Science attitudes	Age	–0.01	0.00	–5.88*	–0.01	0.00	–5.64*
	Education	–0.12	0.01	–9.75*	–0.15	0.01	–12.00*
	Mother’s education	–0.03	0.01	–2.58*	–0.02	0.01	–1.46

Highest education level was set as the baseline for education and mother’s education.

* *p* < .05.

Not surprisingly, education correlated with all four science capital dimensions, showing that respondents with higher education levels had higher science capital. For negative science-related self-efficacy (χ^2^(1) = 7.63, *p* = .006) and early encouragement (χ^2^(1) = 7.07, *p* = .008), the association with education was stronger for men than for women. Finally, when age and education were controlled for, mother’s education was positively associated with the four dimensions of science capital, except for negative science-related self-efficacy for men. Note that even though the regression slopes for mother’s education were not statistically significant for men, they differed from the women’s regression slopes only for the negative science-related self-efficacy (χ^2^(1) = 10.17, *p* = .001).

The regression slopes for the SEM model with age, education, and father’s education as predictors of science capital factors, separately for women and men, are presented in Table 5. Father’s education was positively associated with all four dimensions of science capital. There were no statistically significant differences in the regression slopes between gender groups.

**Table 5.** Regression slopes of the SEM predicting latent factors of science capital with age, education level, and father’s education level with gender as a grouping factor.

Factor	Predictor	Gender group					
		Women			Men		
		Estimate	*SE*	*z*	Estimate	*SE*	*z*
F1: Science-related activities	Age	0.00	0.00	1.76	–0.00	0.00	–1.14
	Education	–0.23	0.02	–14.97*	–0.26	0.02	–14.24*
	Father’s education	–0.05	0.01	–3.62*	–0.04	0.02	–1.86
F2: Negative self-efficacy	Age	0.00	0.00	4.80*	0.01	0.00	11.95*
	Education	0.19	0.01	19.53*	0.23	0.01	19.75*
	Father’s education	0.05	0.01	5.74*	0.05	0.01	4.06*
F3: Early encouragement	Age	–0.00	0.00	–3.28*	–0.01	0.00	–9.61*
	Education	–0.11	0.01	–7.73*	–0.17	0.02	–10.84*
	Father’s education	–0.06	0.02	–3.57*	–0.06	0.02	–3.64*
F4: Science attitudes	Age	–0.01	0.00	–5.69*	–0.01	0.00	–5.29*
	Education	–0.12	0.01	–9.67*	–0.15	0.01	–12.14*
	Father’s education	–0.06	0.01	–5.19*	–0.04	0.01	–2.99*

Highest education level was set as the baseline for education and father’s education.

* *p* < .05.

## Discussion

The present study examined two research questions: (1) what are the dimensions of science capital in adulthood and 2) how do gender, age, education, and parental education relate to science capital? Our results indicated that adult science capital contains four separate but intercorrelated dimensions: science-related self-efficacy and identity, attitudes and dispositions toward science, early support from parents and teachers, and visiting science-related places. These results align well with previous research on science capital among adolescents and youth, which has shown that science-related self-efficacy and identity and attitudes and dispositions toward science are important aspects of science capital (e.g. Archer et al., 2015; Jones et al., 2021). Interestingly, family attitudes and experiences of support during school years emerged as a significant factor in contributing to science capital also in adulthood (Archer et al., 2015; Jones et al., 2021 but see Turnbull et al., 2020). This points to the importance of family attitudes and expectations in forming science capital (Chen et al., 2022). Experiences of encouragement to study science shown by parents or teachers carry over to adulthood, underlining the importance of science teaching that supports students’ science-related self-efficacy and identity and positive attitudes and dispositions toward science. Finally, visiting science-related places emerged as a separate factor, showing that free-choice and informal science learning contexts like science centers and museums play an important role in the formation of science capital in adulthood (Gutwill, 2018).

One should note that the science attitudes and dispositions dimension of SCQ covers a different set of variables than those typically examined in, for example, science communication literature. This literature has often focused on specific concepts such as trust in science and scientists (Nadelson et al., 2014), credibility of science (Hartman et al., 2017), or deference to science (e.g. Howell et al., 2020). The items included in the science attitudes and dispositions dimension tapped into how respondents perceive the usefulness of science in their daily lives or society in general. The SCQ helps to disentangle how these types of science attitudes are linked with self-perceptions in relation to science, early experiences of encouragement to study science at school and at home, and participation in science-related activities. Future studies could explore how these different dimensions of science capital are related to the other types of science attitudes.

The data supported a model in which the four dimensions of science capital are distinct yet correlated. The high correlation between early encouragement to study science and science-related self-efficacy shows that the early experiences at school and at home continue to have an impact on one’s perception of the self in relation to science in adulthood. On the other hand, the relatively low correlation between science attitudes and science-related activities suggests that having a positive disposition toward science does not necessarily mean that one actively participates in science-related activities. These findings suggest that to understand science capital and its accumulation and impact on people’s lives, the different dimensions should be examined separately. Our results are in line with Jones et al. (2021), who also found that a four-factor solution without a second-order factor was the best fit to their data. The practical implication of this finding is that when measuring science capital, one should measure the different dimensions separately. This contrasts with the approach of Archer et al. (2015), who have used a single science capital index as a predictor of, for example, science aspirations among youth. However, as the dimensions are correlated, a single index may be sufficient to represent science capital on the condition that the index is composed of a balanced set of items from the different dimensions.

Our second research question concerned the distribution of science capital in society. We found that age, education, and parents’ education all predicted science capital in the adult population. As for the effect of age on different aspects of science capital, older respondents had lower levels of science-related self-efficacy, had experienced less early support, and showed more negative attitudes toward science than younger respondents. However, no effect of age was observed on participation in science-related activities. These findings reflect the findings of recent international surveys (e.g. Eurobarometer, 2021), which show that while older people may be interested in science, they tend to feel that science is too complicated for them to understand and has little relevance to their daily lives. In other words, older people tend to have lower science-related self-efficacy, and their dispositions toward science are not as positive as those of younger people. Our results also indicate that they may have experienced less support to study natural sciences during their school years.

Not surprisingly, highly educated respondents had higher science capital across all four dimensions of science capital than the less-educated respondents (e.g. Noy and O’Brien, 2019). Furthermore, parents’ education level was a significant predictor of all four dimensions of science capital in adulthood, further pinpointing the importance of family background in gaining science capital (Armila et al., 2018; Chen et al., 2022; Gonsalves et al., 2021).

As for differences between gender groups, we examined the association between background variables on the different dimensions of science capital separately for men and women. This was because even though the data supported configural invariance between gender groups, that is similarity in which items load onto which latent factors, the factor loadings were not similar across the groups, making it impossible to directly compare men and women. The results of the SEMs showed that age and education were stronger predictors of science-related self-efficacy and experienced early encouragement for men than for women, indicating that the observed “drop” in science-related self-efficacy and early support in older age was particularly steep for men, and that the difference in science capital between less-educated and highly educated respondents was greater for men than for women. On the other hand, mother’s education had a weaker association with science-related self-efficacy for men than for women, showing that when respondents’ age and education were controlled for, men’s science-related self-efficacy was not associated with maternal education. These findings shed new light on the nature of science capital and its accumulation. Previous research has focused on direct comparisons between gender groups in the amount of science capital (Archer et al., 2015; Moote et al., 2021), and little attention has been paid to understanding whether science capital is a similar construct for different genders or not. Prior research has demonstrated that, for example, women have lower science-related self-efficacy and a “not a science person” identity even if they are highly educated (see Kelly et al., 2019; Turnbull et al., 2020). Thus, age or education may not have as strong an impact on women’s science capital as it has for men.

### Limitations of the current study

Although the present study helps to characterize the dimensions and distribution of science capital in adulthood, it naturally has its limitations. The first limitation is the nature of our sample. Despite the initial sample being representative of the Finnish population, females were overrepresented in the final sample. This might reflect women’s greater interest in scientific research. Moreover, due to the small percentage of Finns belonging to language minority groups, it was not possible to conduct analyses on the impact of language background on science capital—the subsamples were too small. In the future, targeted samples of underrepresented groups, including language minorities, should be recruited to examine how cultural and linguistic background is reflected in the build-up of science capital.

Second, although we carefully planned and piloted our survey questions, some important aspects of science capital might have been overlooked. As in many previous studies (Archer et al., 2015; DeWitt et al., 2016), we did not include a direct measure of science knowledge in the SCQ and asked participants to estimate their own science literacy. Understanding how the different dimensions of science capital identified in the present study are associated with actual scientific knowledge and understanding of scientific concepts is important and should be studied in the future. Moreover, the SCQ was based on previous SCQs designed for adolescents and youth (Archer et al., 2015; DeWitt et al., 2016) and we might have missed some aspects that are relevant for accumulating science capital in adulthood. Future studies should also use qualitative methods, like interviews, to study the more detailed nuances of cultural and social science capital and how they are produced and reproduced in adulthood.

Previous research suggests that political ideology and religious beliefs have an impact on how people accept or reject scientific explanations and policies (e.g. Allum et al., 2014; Blank and Shaw, 2015), suggesting that these factors might be important for forming science capital. In the present study, we did not consider these factors. Future studies should investigate in more detail whether political and religious beliefs are associated with the formation of science capital in general, or if they mainly impact science attitudes on specific, belief-relevant topics (see Blank and Shaw, 2015).

Finally, it should be noted that these data were collected in Finland, a relatively small northern European welfare state that has a multiparty political system; has two official state-recognized churches that the majority of inhabitants belong to (66% in 2022); offers free education from preschool to the university level (Välimaa, 2021); and where general attitudes toward science are positive (Eurobarometer, 2021). Interestingly, socio-demographic factors were associated with dimensions of science capital also in the Finnish context, showing how social inequalities manifest in the global science domain. However, the generalizability of the present results to other societal contexts should be considered with some caution.

## Conclusions

The present study shows that science capital in adulthood is based on science-related self-efficacy and identity, attitudes and dispositions toward science, support from family and teachers during school years, and visits to science-related places. Instead of focusing on what kind of (or how much) science-related knowledge people hold, the concept of science capital puts focus on understanding the factors that influence an individual’s approach to science and scientific information. It seems that while formal education plays a significant role in the build-up of science capital in adulthood, many of the dimensions are related to experiences outside of formal education. The current findings point to mechanisms that produce social inequalities and provide useful information for designing actions and policies that support the accumulation of science capital in society. Improving science capital is important, as it may have political, economic, and social consequences at the micro and macro levels of society (see Laugksch, 2000; Yacoubian, 2018). For individuals, science capital provides means for making informed decisions in their daily lives, such as deciding on whether to take a vaccine recommended by health officials. It may provide more or novel opportunities in work life and increase the opportunities to enjoy science as a form of human culture. At the macro level, the public with high science capital can be expected to support science domains and contribute to democratic decision-making, which is crucial for finding solutions to global problems such as climate change. However, the current evidence is correlational, and the efficacy of the measures suggested below should be tested experimentally in the future.

First, while targeting science literacy skills at school is important, investing in school pedagogies that build up interest and engagement in science, raise understanding of the importance of science in everyday life, and aim at breaking the stereotypical views of whom science belongs to would help in building science self-efficacy and positive attitudes toward science from an early stage (Claussen and Osborne, 2013). The support from teachers is especially important in building positive science self-efficacy: teachers should give positive feedback and encouragement, and provide individually supportive activities to all students, not only the motivated and high-achieving pupils in the classroom.

Second, there is potential in informal and free-choice science learning to influence science capital. For example, 54% of EU citizens report that they would like to learn more about scientific developments via public services like libraries or museums (Eurobarometer, 2021), indicating that the general public is willing to engage in lifelong science learning. Helping institutions such as public libraries, museums, and science centers to develop actions that would reach wider audiences is potentially an effective measure to build science capital (Gutwill, 2018).

Third, we need to invest in science communication that is trustworthy, understandable, and reaches diverse audiences, as that would build and support positive attitudes toward science and scientific information. We suggest that these measures would increase citizens’ science capital, which would help in closing the gap between scientific knowledge and public acceptance of it, and support citizens and politicians in making better decisions toward solving the significant challenges our society is currently facing (Sinatra and Hofer, 2016, 2021).

## Supplemental Material

sj-docx-1-pus-10.1177_09636625241310756 – Supplemental material for Science capital: Results from a Finnish population survey Supplemental material, sj-docx-1-pus-10.1177_09636625241310756 for Science capital: Results from a Finnish population survey by Johanna K. Kaakinen, Sari Havu-Nuutinen, Tuomo Häikiö, Hanna Julku, Teija Koskela, Mirjamaija Mikkilä-Erdmann, Milla Pihlajamäki, Daria Pritup, Kirsi Pulkkinen, Katri Saarikivi, Jaana Simola and Valtteri Wikström in Public Understanding of Science
