# Supplementary material for: Science capital: Results from a Finnish population survey
Source: Public Underst Sci. 2025 Feb 14;34(6):770–90. doi: 10.1177/09636625241310756 (PMC12274560; doi:10.1177/09636625241310756)
Supplement: sj-docx-1-pus-10.1177_09636625241310756 – Supplemental material for Science capital: Results from a Finnish population survey [file sj-docx-1-pus-10.1177_09636625241310756.docx]

**Supplemental material**

**Science Capital: Results from a Finnish Population Survey**

Johanna K. Kaakinen^1,2^, Sari Havu-Nuutinen^3^, Tuomo Häikiö^1^, Hanna Julku^4^, Teija Koskela^5^, Mirjamaija Mikkilä-Erdmann^5^, Milla Pihlajamäki^4^, Daria Pritup^1^, Kirsi Pulkkinen^6^, Katri Saarikivi^1^, Jaana Simola^4^, & Valtteri Wikström^1^

^1^ Department of Psychology and Speech-Language Pathology, University of Turku

^2^ INVEST Research flagship, University of Turku

^3^ School of Applied Educational Science and Teacher Education, Philosophical Faculty,

University of Eastern Finland

^4^ Department of Education, University of Helsinki

^5^ Department of Teacher Education, University of Turku

^6^ R&D Services, Metropolia University of Applied Sciences

This supplement contains information about the SCQ items and the statistical analyses reported in the article “Science Capital: Results form a Finnish Population Survey”. The supplement contains the following sections: 1. Description of the SCQ items and their sources; 2. Representativeness of the sample; 3. Data preprocessing; 4. Statistical analyses, missing data and imputation; 5. Exploratory factor analysis; 6. Confirmatory factor analysis; 7. Testing for metric invariance; and 8. References.

1. **Description of the SCQ items**

**Table A. Finnish Science Capital Questionnaire (SCQ) questions, their sources, categories as identified in previous research, and loading on factors found in the present study.**

| **Question** | **Item Source** | **Original Item Category** | **Factor** |
| --- | --- | --- | --- |
| Do you have relatives, friends or colleagues, who work with/in science? | A & PAS | Knowing people in science related roles | F2 |
| Do you speak about things related to science with other people? How often? | M & PAS | Conversation | F2 |
| **How often do you usually visit the following places or participate in the following activities in your free time?** Think about the time before the coronavirus pandemic. |  |  |  |
| Places related to science (e.g., observatories or botanical gardens) | A & ASP | Valuing museums/museum experiences | F1 |
| Art museum | A & ASP | Valuing museums/museum experiences | F1 |
| Other museum (not a science or art museum, e.g., Ainola, local or arts and crafts museum) | A & ASP | Valuing museums/museum experiences | F1 |
| Science or technology museum | A | Informal science activities | F1 |
| Science centre | A | Informal science activities | NI |
| Literature event | ASP | NR | F1 |
| Planetarium | A | Informal science activities | NI |
| Lecture, talk or presentation (e.g., webinar) related to science or technology outside of school or work | PAS & M | Leisure activity | F1 |
| National park or other nature site | A | Informal science activities | F1 |
| Zoo or aquarium | A | Informal science activities | NI |
|  |  |  |  |
|  |  |  |  |
| **Think of your time in school. How much do you agree with the following statements? Please mark one response per line.**In this section, “science” means natural science subjects taught in school: physics, chemistry, biology and geography. |  |  |  |
| My parents or my guardians emphasised that science would be beneficial for me in the future. | A | Utility of science qualifications | F3 |
| My parents or my guardians thought it was important for me to study science in school. | A | Utility of science qualifications | F3 |
| A significant adult (such as parent or teacher) encouraged me to continue science studies after primary and secondary compulsory education. | A | Science teachers and lessons | F3 |
| My parents or my guardians were interested in science. | A | Family attitudes | F3 |
| My teachers emphasised that science would be beneficial for me in the future. | A | Science teachers and lessons | F3 |
| My teachers thought I was gifted in science. | A & ASP | Science teachers and lessons | F3 |
| **How much do you agree with the following statements?** |  |  |  |
| Young people’s interest in science is essential for our future welfare. | ASP | NR | F4 |
| It’s important for our society that young people understand science. | A | Valuing science and scientist | F4 |
| Science has so much significance in our lives that everyone should be interested in it. | ASP | NR | F4 |
| Science is important for understanding the world. | ASP | NR | F4 |
| Scientific knowledge is useful in my daily life. | ASP & PAS | NR | F4 |
| The scientific knowledge I learned in school has been useful in my daily life. | ASP & PAS | Knowledge about the transferability of science | F4 |
| The next generation has more opportunities for work due to science and technology. | ASP | NR | NI |
| I’m interested in scientific research and findings. | M | Self-report for interest | F4 |
| The mathematics I learned in school has been useful in my daily life. | ASP | NR |  |
| I don’t think I’m smart enough to understand science. | ASP | Self-efficacy in science | F2 |
| I don’t think I’m smart enough to understand technology. | ASP | Self-efficacy in science | F2 |
| I don’t actually know what a scientist does. | ASP | NR | F2 |
| I don’t actually know what an engineer does. | ASP | NR | F2 |
| Science is not for me. | ASP & PAS | Participation with science outside of learning contexts, and consumption of science media | F2 |
| I can well understand scientific terminology, such as hypothesis, theory, experiment and clinical trial. | ASP & PAS | Scientific literacy | F2 |
| I’m well informed about science, scientific research and their developments. | ASP & PAS | Scientific literacy | F2 |
| School put me off science. | ASP & PAS | Perceptions of school science | F2 |
| I would feel comfortable in places where science is discussed and practiced, such as laboratories, science centres, and industrial environments. | ASP & PAS | Science-related attitudes, values, and dispositions | F2 |
| I follow news about new technologies. | M | News interest | F2 |

*Note.* Item source: A = Archer et al. (2015), M = Motta et al. (2021), ASP = ASPIRES project, PAS = Public attitudes to science 2019. Original item category: NR = not reported in publications. Factor: F1=Science-related activities, F2=Negative self-efficacy, F3=Early encouragement, F4=Science attitudes, NI = not included in the final SCQ.

1. **Representativeness of the sample**

We examined how representative the final sample was of the original larger sample by comparing the distributions of the background variables in the final sample (N = 1,572) to the original sample (N = 8,500). For gender, the information in the population sample was stored as a binary variable, so the "other" responses were not considered in this comparison. In the final sample, females were over-represented, χ^2^(1) = 16.38, *p* < .001. The distributions of age (χ^2^(12) = 8.51, *p* = .744), language (χ^2^(1) = 0.02, *p* = .901) and geographical region (χ^2^(17) = 1.22, *p* = 1) did not differ from the original sample.

1. **Data preprocessing**

Each questionnaire contained a unique ID code that was used to keep track of participants who had responded to the survey. We discarded responses that did not match the original participant ID codes (11 responses) and removed duplicates from the data. Participants who demonstrated careless responding were excluded on two steps. First, we excluded participants who had missing data for more than 1/3 of the science capital questionnaire items (55 responses, 3.47% of the data). We then excluded respondents who had selected the same response option for 95% of the Likert-scale items (13 responses, 0.85% of the data). The final sample in the analyses was N=1,515.

Responses to the item "Do you have relatives, friends or colleagues who work in science?" were recoded so that "I do not know" answers (n=191) were combined with "no" (n=722), resulting in a binary variable (1 = yes, 0 = no or I do not know). Two items regarding the frequency of visits to science-related places (Science laboratory or other science environment and Science festival or fair) had very skewed distributions (62% and 65% of the respondents, respectively, reported that they never visit these places), and these items were left out from the analyses. Also, the Other option of the visits to science-related places was left out of the analyses, as it required an open-ended response and had a lot of missing data (53%).

1. **Statistical analyses, missing data and imputation**

The data were analyzed in R (version 4.1.3; R Core Team, 2022), using the *psych* (version 2.2.9, Revelle, 2022), *lavaan* (version 0.6-11; Rosseel, 2012), and *semTools* (version 0.5-6; Jorgensen et al., 2022) packages.

All analyses were conducted for non-imputed data with missing values, as well as for multiple imputed datasets for the science capital survey items with the *mifa* (version 0.2.0; Nassiri et al., 2021) and *mice* (3.14.0; Van Buuren and Groothuis-Oudshoorn, 2011) packages for R. Note that we only imputed values for the science capital items, not the background variables. As the analyses with the imputed datasets produced very similar results to those obtained with non-imputed data, we report the results of the analyses conducted with non-imputed data.

1. **Exploratory factor analysis (EFA)**

The appropriate factor stucture for the SCQ was first examined by conducting an EFA on a random sample that contained 50% of the data (N=757). For the EFA, we used a polychoric correlation matrix provided by the mixedCor() function in the *psych* package, and pairwise missing values were excluded. The potential number of factors supported by the data was examined with tests provided by the nfactors() function.

The number of factors to be extracted was based on different indexes (VSS complexity 1, VSS complexity 2, Velicer MAP, empirical BIC, sample size adjusted BIC) and interpretation of the scree plot. The VSS complexity 1 indicated one factor, VSS complexity 2 three factors, the Velicer MAP four factors, empirical BIC seven, and sample size adjusted BIC 12 factors. We initially fit the one, three, four and seven-factor solutions to the data as they could be supported by the theoretical framework and previous empirical studies on science capital.

The EFAs were conducted using weighted least squares estimation and oblimin rotation. The factor models for the one, three, four, and seven-factor models are presented in Tables B-E. We compared the different solutions by looking at the proportion of variance explained and item loadings, and decided to retain the four-factor solution as our final model because it explained a relatively high proportion of variance in the data (44%) and provided a theoretically plausible interpretation of the factors. All items showed factor loadings > .33 (see Table D). Factor 1 included items related to negative science self-efficacy (e.g., "I don't think I'm smart enough to understand science"), and it explained 12% of the total variance. Items that loaded on Factor 2 were all related to the frequency of visiting science-related places, and it explained an additional 12% of the total variance. Factor 3 consisted of items on science attitudes and dispositions (e.g., "It is essential for our future that young people are interested in science"), and it explained an additional 10% of the total variance. Factor 4 included items related to support from the teachers and parents when the respondent was at school, and it accounted for 10% of the total variance. The factors showed moderate correlations, |r| = .32 - .45. In comparison to the three-factor model, the four-factor model separated items related to science attitudes and dispositions from negative self-efficacy, while retaining the factors for visiting science-related places and support from parents and teachers as separate latent constructs. In comparison to the seven-factor model, it provided a more comprehensive (all items fit to the model) and interpretable (not too many cross-loading of items) solution.

**Table B. EFA factor loadings in the one-factor solution.**

| **Item** | **Factor loading** |
| --- | --- |
| Do you speak about things related to science with other people? How often? | 0.67 |
| I can well understand scientific terminology, such as hypothesis, theory, experiment and clinical trial. | 0.66 |
| I’m interested in scientific research and findings. | 0.65 |
| Science is not for me. | -0.64 |
| I’m well informed about science, scientific research and their developments. | 0.64 |
| Scientific knowledge is useful in my daily life. | 0.63 |
| I don’t think I’m smart enough to understand science. | -0.62 |
| I would feel comfortable in places where science is discussed and practiced, such as laboratories, science centres, and industrial environments. | 0.61 |
| Science is important for understanding the world. | 0.61 |
| I don’t actually know what a scientist does. | -0.59 |
| How often do you usually visit science or technology museums in your free time? | 0.59 |
| Do you have relatives, friends or colleagues, who work with/in science? | 0.59 |
| The scientific knowledge I learned in school has been useful in my daily life. | 0.58 |
| I don’t think I’m smart enough to understand technology. | -0.56 |
| How often do you usually visit places related to science (e.g., observatories or botanical gardens) in your free time? | 0.54 |
| How often do you usually visit lectures, talks or webinars related to science or technology in your free time? | 0.54 |
| I follow news about new technologies. | 0.54 |
| My parents or my guardians thought it was important for me to study science in school. | 0.53 |
| I don’t actually know what an engineer does. | -0.50 |
| My parents or my guardians emphasised that science would be beneficial for me in the future. | 0.50 |
| My teachers thought I was gifted in science. | 0.49 |
| How often do you usually visit art museums in your free time? | 0.49 |
| My parents or my guardians were interested in science. | 0.48 |
| A significant adult (such as parent or teacher) encouraged me to continue science studies after primary and secondary compulsory education. | 0.48 |
| Young people’s interest in science is essential for our future welfare. | 0.48 |
| How often do you usually visit science centres in your free time? | 0.48 |
| It’s important for our society that young people understand science. | 0.46 |
| School put me off science. | -0.46 |
| My teachers emphasised that science would be beneficial for me in the future. | 0.46 |
| How often do you usually visit national parks or other nature sites in your free time? | 0.44 |
| Science has so much significance in our lives that everyone should be interested in it. | 0.41 |
| How often do you usually visit other museums (e.g., local or arts and crafts museums) in your free time? | 0.38 |
| The mathematics I learned in school has been useful in my daily life. | 0.36 |
| How often do you usually visit literature events in your free time? | 0.29 |
| How often do you usually visit planetariums in your free time? | 0.28 |
| The next generation has more opportunities for work due to science and technology. | 0.24 |
| How often do you usually visit zoos or aquariums in your free time? | 0.16 |

Note. Explains 27% of the variance.

**Table C. EFA factor loadings and the correlations between factors in the three-factor solution.**

|  | | **Factor loading** | | |  |
| --- | --- | --- | --- | --- | --- |
| **Item** | Factor 1 | | Factor  2 | Factor 3 | |
| Science is not for me. | -0.74 | | 0.02 | 0.06 | |
| I don't think I'm smart enough to understand science. | -0.72 | | 0.04 | 0.04 | |
| I don't think I'm smart enough to understand technology. | -0.67 | | 0.09 | 0.00 | |
| I'm interested in scientific research and findings. | 0.65 | | 0.08 | -0.01 | |
| I can well understand scientific terminology, such as hypothesis, theory, experiment and clinical trial. | 0.64 | | 0.03 | 0.06 | |
| I'm well informed about science, scientific research and their developments. | 0.63 | | 0.06 | 0.00 | |
| I don't actually know what a scientist does. | -0.62 | | -0.02 | 0.00 | |
| I would feel comfortable in places where science is discussed and practiced, such as laboratories, science centres, and industrial environments. | 0.59 | | -0.02 | 0.12 | |
| Scientific knowledge is useful in my daily life. | 0.58 | | 0.08 | 0.04 | |
| School put me off science. | -0.57 | | 0.10 | 0.00 | |
| Science is important for understanding the world. | 0.57 | | 0.05 | 0.06 | |
| Do you speak about things related to science with other people? How often? | 0.57 | | 0.21 | -0.01 | |
| Young people's interest in science is essential for our future welfare. | 0.55 | | -0.03 | -0.02 | |
| I follow news about new technologies. | 0.55 | | 0.02 | 0.02 | |
| I don't actually know what an engineer does. | -0.53 | | 0.03 | -0.04 | |
| The scientific knowledge I learned in school has been useful in my daily life. | 0.51 | | 0.03 | 0.11 | |
| It's important for our society that young people understand science. | 0.49 | | 0.00 | 0.00 | |
| Science has so much significance in our lives that everyone should be interested in it. | 0.48 | | -0.04 | -0.03 | |
| Do you have relatives, friends or colleagues, who work with/in science? | 0.44 | | 0.23 | 0.04 | |
| The mathematics I learned in school has been useful in my daily life. | 0.36 | | -0.08 | 0.11 | |
| The next generation has more opportunities for work due to science and technology. | 0.24 | | -0.01 | 0.04 | |
| How often do you usually visit the following places or participate in the following activities in your free time? |  | |  |  | |
| Places related to science (e.g., observatories or botanical gardens) | 0.04 | | 0.75 | 0.03 | |
| Art museums | 0.05 | | 0.75 | -0.07 | |
| Other museums (e.g., local or arts and crafts museums) | -0.11 | | 0.73 | 0.01 | |
| Science or technology museums | 0.11 | | 0.67 | 0.07 | |
| Science centres | 0.04 | | 0.64 | 0.02 | |
| Literature events | -0.07 | | 0.57 | -0.03 | |
| Planetariums | -0.06 | | 0.52 | 0.00 | |
| Lectures, talks or webinars related to science | 0.25 | | 0.45 | 0.01 | |
| Zoos or aquariums | -0.19 | | 0.45 | 0.06 | |
| National parks or other nature sites | 0.10 | | 0.44 | 0.08 | |
| My parents or guardians emphasised that science would be beneficial for me in the future. | -0.06 | | -0.02 | 0.93 | |
| My parents or guardians thought it was important for me to study science in school. | 0.01 | | 0.03 | 0.81 | |
| A significant adult (such as parent or teacher) encouraged me to continue science studies. | 0.02 | | 0.01 | 0.73 | |
| My parents or guardians were interested in science. | 0.04 | | 0.01 | 0.71 | |
| My teachers emphasised that science would be beneficial for me in the future. | 0.03 | | 0.05 | 0.63 | |
| My teachers thought I was gifted in science. | 0.23 | | -0.06 | 0.51 | |
|  | | Factor correlations | | | |
| Factor 1 | 1.00 | | .45 | .45 | |
| Factor 2 |  | | 1.00 | .30 | |
| Factor 3 |  | |  | 1.00 | |
|  | | Proportion of variance | | | |
|  | 20% | | 11% | 9% | |

*Note*. Explains 40% of the variance.

**Table D. EFA factor loadings and the correlations between factors in the four-factor solution.**

|  | **Factor loading** | | | |
| --- | --- | --- | --- | --- |
| **Item** | Factor 1 | Factor 2 | Factor 3 | Factor 4 |
| I don't think I'm smart enough to understand science. | 0.86 | -0.01 | 0.06 | 0.02 |
| I don't think I'm smart enough to understand technology. | 0.84 | 0.04 | 0.10 | -0.03 |
| I don't actually know what a scientist does. | 0.58 | -0.07 | -0.08 | -0.03 |
| I don't actually know what an engineer does. | 0.51 | -0.01 | -0.06 | -0.07 |
| Science is not for me. | 0.49 | -0.04 | -0.32 | 0.02 |
| I can well understand scientific terminology, such as hypothesis, theory, experiment and clinical trial. | -0.46 | 0.08 | 0.23 | 0.09 |
| I'm well informed about science, scientific research and their developments. | -0.45 | 0.11 | 0.23 | 0.04 |
| School put me off science. | 0.38 | 0.07 | -0.26 | -0.02 |
| I would feel comfortable in places where science is discussed and practiced, such as laboratories, science centres, and industrial environments. | -0.38 | 0.02 | 0.26 | 0.15 |
| I have relatives, friends or colleagues, who work with/in science. | -0.36 | 0.27 | 0.10 | 0.06 |
| I speak about things related to science with other people. | -0.35 | 0.25 | 0.26 | 0.02 |
| I follow news about new technologies. | -0.34 | 0.06 | 0.26 | 0.05 |
| I visit places related to science (e.g., observatories or botanical gardens) in my free time. | -0.01 | 0.76 | 0.01 | 0.03 |
| I visit art museums in my free time. | 0.03 | 0.76 | 0.06 | -0.07 |
| I visit other museums (e.g., local or arts and crafts museums) in my free time. | 0.11 | 0.73 | -0.04 | 0.00 |
| I visit science or technology museums in my free time. | -0.11 | 0.69 | -0.03 | 0.08 |
| I visit science centres in my free time. | -0.02 | 0.66 | 0.00 | 0.02 |
| I visit literature events in my free time. | 0.07 | 0.57 | -0.03 | -0.04 |
| I visit planetariums in my free time. | 0.02 | 0.52 | -0.07 | -0.01 |
| I visit lectures, talks or webinars related to science or technology in my free time. | -0.20 | 0.48 | 0.05 | 0.02 |
| I visit national parks or other nature sites in my free time. | -0.07 | 0.46 | 0.02 | 0.08 |
| I visit zoos or aquariums in my free time. | 0.14 | 0.45 | -0.08 | 0.05 |
| Young people's interest in science is essential for our future welfare. | 0.08 | -0.02 | 0.78 | -0.01 |
| It's important for our society that young people understand science. | 0.05 | 0.01 | 0.65 | 0.02 |
| Science has so much significance in our lives that everyone should be interested in it. | 0.03 | -0.03 | 0.62 | -0.02 |
| Science is important for understanding the world. | -0.14 | 0.08 | 0.52 | 0.09 |
| Scientific knowledge is useful in my daily life. | -0.18 | 0.12 | 0.48 | 0.06 |
| The scientific knowledge I learned in school has been useful in my daily life. | -0.15 | 0.06 | 0.44 | 0.14 |
| The next generation has more opportunities for work due to science and technology. | 0.11 | -0.01 | 0.41 | 0.04 |
| I'm interested in scientific research and findings. | -0.32 | 0.13 | 0.39 | 0.02 |
| The mathematics I learned in school has been useful in my daily life. | -0.08 | -0.07 | 0.35 | 0.13 |
| My parents/guardians emphasised that science would be beneficial for me in the future. | 0.07 | -0.03 | -0.01 | 0.94 |
| My parents/guardians thought it was important for me to study science in school. | 0.00 | 0.03 | 0.00 | 0.81 |
| A significant adult (e.g., parent or teacher) encouraged me to continue science studies. | -0.08 | 0.02 | -0.07 | 0.73 |
| My parents/guardians were interested in science. | 0.00 | 0.01 | 0.03 | 0.72 |
| My teachers emphasised that science would be beneficial for me in the future. | 0.07 | 0.05 | 0.10 | 0.63 |
| My teachers thought I was gifted in science. | -0.24 | -0.04 | -0.01 | 0.53 |
|  | Factor correlations | | | |
| Factor 1 | 1.00 | -.35 | -.45 | -.36 |
| Factor 2 |  | 1.00 | .36 | .32 |
| Factor 3 |  |  | 1.00 | .34 |
| Factor 4 |  |  |  | 1.00 |
|  | Proportion of variance | | | |
|  | 12% | 12% | 10% | 10% |

*Note*. Explains 44% of the variance.

**Table E. EFA factor loadings and the correlations between factors in the seven-factor solution.**

|  | **Factor loading** | | | | | | |
| --- | --- | --- | --- | --- | --- | --- | --- |
| **Item** | Factor 1 | Factor 2 | Factor 3 | Factor 4 | Factor 5 | Factor 6 | Factor 7 |
| My parents or guardians… |  |  |  |  |  |  |  |
| emphasised that science would be beneficial for me in the future. | 0.93 | 0.00 | -0.01 | -0.06 | 0.06 | -0.07 | -0.01 |
| thought it was important for me to study science in school. | 0.82 | -0.02 | 0.06 | 0.00 | 0.03 | -0.04 | -0.03 |
| were interested in science. | 0.73 | 0.03 | 0.04 | 0.09 | 0.02 | -0.03 | -0.05 |
| A significant adult (e.g. parent or teacher) encouraged me to continue science studies. | 0.71 | -0.03 | -0.09 | 0.04 | -0.11 | 0.09 | 0.11 |
| My teachers… |  |  |  |  |  |  |  |
| emphasised that science would be beneficial for me in the future. | 0.61 | 0.06 | 0.04 | -0.05 | 0.05 | 0.14 | 0.02 |
| thought I was gifted in science. | 0.47 | -0.10 | -0.07 | 0.08 | -0.20 | 0.34 | 0.00 |
| I don't think I'm smart enough to understand technology. | -0.02 | 0.91 | 0.09 | 0.01 | 0.01 | 0.06 | -0.09 |
| I don't think I'm smart enough to understand science. | 0.03 | 0.79 | -0.04 | -0.11 | 0.05 | -0.04 | 0.01 |
| I don't actually know what an engineer does. | -0.06 | 0.57 | -0.10 | 0.14 | -0.11 | -0.10 | 0.03 |
| I don't actually know what a scientist does. | -0.04 | 0.55 | -0.24 | -0.01 | -0.07 | -0.10 | 0.14 |
| I follow news about new technologies. | 0.04 | -0.25 | -0.16 | 0.24 | 0.20 | 0.05 | 0.24 |
| How often do you usually visit the following places or participate in the following activities in your free time? |  |  |  |  |  |  |  |
| Art museums | 0.00 | -0.02 | 0.80 | 0.01 | 0.09 | -0.05 | 0.05 |
| Other museums (e.g., local or arts and crafts museums) | 0.05 | 0.01 | 0.68 | -0.11 | 0.02 | -0.01 | 0.15 |
| Literature events | -0.02 | 0.12 | 0.52 | 0.08 | -0.10 | 0.10 | 0.07 |
| Places related to science (e.g., observatories or botanical gardens). | 0.06 | -0.03 | 0.48 | 0.06 | 0.02 | 0.03 | 0.35 |
| National parks or other nature sites | 0.06 | -0.17 | 0.36 | 0.28 | -0.09 | 0.20 | -0.12 |
| Lectures, talks or webinars related to science or technology | 0.09 | -0.02 | 0.34 | 0.11 | -0.03 | 0.08 | 0.14 |
| I'm interested in scientific research and findings. | 0.03 | -0.04 | 0.34 | 0.27 | -0.08 | 0.14 | 0.15 |
| I'm well informed about science, scientific research and their developments. | 0.04 | -0.01 | -0.07 | 0.65 | 0.18 | 0.03 | 0.13 |
| Do you speak about things related to science with other people? How often? | 0.04 | -0.17 | 0.00 | 0.53 | 0.03 | 0.09 | 0.05 |
| I can well understand scientific terminology, such as hypothesis, theory, experiment and clinical trial. | 0.03 | -0.07 | 0.18 | 0.52 | 0.03 | 0.13 | 0.01 |
| Science is not for me. | 0.12 | -0.26 | 0.18 | 0.39 | 0.09 | 0.05 | -0.16 |
| I would feel comfortable in places where science is discussed and practiced, such as laboratories, science centres, and industrial environments. | -0.01 | 0.31 | -0.01 | -0.39 | -0.21 | -0.02 | 0.00 |
| Young people's interest in science is essential for our future welfare. | 0.15 | -0.25 | -0.08 | 0.25 | 0.16 | 0.09 | 0.10 |
| It's important for our society that young people understand science. | 0.01 | 0.04 | 0.06 | 0.06 | 0.66 | 0.14 | -0.05 |
| Science has so much significance in our lives that everyone should be interested in it. | 0.06 | -0.02 | 0.06 | 0.06 | 0.62 | -0.01 | -0.01 |
| The next generation has more opportunities for work due to science and technology. | 0.01 | -0.01 | -0.04 | 0.07 | 0.57 | 0.04 | 0.05 |
| Science is important for understanding the world. | 0.04 | -0.02 | -0.06 | -0.16 | 0.42 | 0.10 | 0.12 |
| The scientific knowledge I learned in school has been useful in my daily life. | 0.12 | -0.03 | 0.09 | 0.30 | 0.38 | 0.06 | -0.03 |
| The mathematics I learned in school has been useful in my daily life. | 0.04 | 0.05 | 0.01 | 0.13 | 0.09 | 0.65 | 0.04 |
| Scientific knowledge is useful in my daily life. | 0.03 | -0.09 | -0.09 | -0.20 | 0.18 | 0.52 | 0.07 |
| School put me off science. | 0.00 | -0.03 | 0.07 | 0.16 | 0.21 | 0.45 | 0.06 |
| I'm interested in scientific research and findings. | 0.03 | 0.32 | -0.04 | 0.04 | -0.09 | -0.38 | 0.09 |
| How often do you usually visit the following places or participate in the following activities in your free time? |  |  |  |  |  |  |  |
| Science centres | 0.01 | -0.01 | 0.15 | 0.11 | 0.01 | 0.03 | 0.63 |
| Planetariums | -0.04 | -0.03 | 0.04 | -0.04 | -0.02 | 0.05 | 0.62 |
| Science or technology museums | 0.09 | -0.13 | 0.28 | 0.08 | 0.02 | 0.00 | 0.51 |
| Zoos or aquariums | 0.04 | 0.04 | 0.08 | -0.12 | 0.00 | 0.01 | 0.46 |
|  | **Factor correlations** | | | | | | |
| Factor 1 | 1.00 | -.31 | .21 | .29 | .23 | .36 | .20 |
| Factor 2 |  | 1.00 | -.19 | -.48 | -.27 | -.39 | -.18 |
| Factor 3 |  |  | 1.00 | .30 | .19 | .20 | .43 |
| Factor 4 |  |  |  | 1.00 | .35 | .40 | .19 |
| Factor 5 |  |  |  |  | 1.00 | .38 | .11 |
| Factor 6 |  |  |  |  |  | 1.00 | .16 |
| Factor 7 |  |  |  |  |  |  | 1.00 |
|  | **Proportion of variance** | | | | | | |
|  | 10% | 9% | 8% | 7% | 6% | 5% | 5% |

*Note.* Explains 50% of the variance.

1. **Confirmatory factor analysis**

We tested the fit of the four-factor model suggested by EFA by conducting a CFA with four single-order latent factors on the other 50% of the data (n = 758). Note that the CFA was fitted to a different data than the EFA. The CFA was computed using the *lavaan* package for R using diagonally weighted least squares estimation and NLMINB optimization.

Modification indexes suggested that the error terms of three pairs of very similarly worded items correlated, and they were released. The final model estimates are presented in Table F. The model fit indices indicated a good fit of the model to the data (Hu and Bentler, 1999): CFI = .979, TLI = .977, RMSEA = .070, 90%CI for RMSEA = [.067,.073], SRMR = .070.

To examine whether there is a single latent construct underlying SCQ scores or if a model with correlated dimensions better fits the data, we compared two CFA models: one with only first-order factors corresponding to different facets of science capital, and another model with a second-order factor representing a latent factor for science capital. The models were compared with a likelihood ratio test (LRT) using the lavTestLRT() function.

The model estimates for the CFA with a second-order factor are presented in Table G. The model fit indices indicated a good fit of the model to the data (Hu and Bentler, 1999), CFI = .978, TLI = .977, RMSEA = .070, 90%CI for RMSEA = [.067, .073], SRMR = .070. However, the likelihood ratio test indicated that this model was worse fit to the data than the model without the second-order factor, χ2(2) = 39.39, p < .001. Thus, the model with four single-order latent factors was retained as the final model.

Finally, the four-factor model was fitted to the full data. Two items, one from the first factor (“Zoos or aquariums”, λ = .329) and one from the fourth factor (“The next generation has more opportunities for work due to science and technology.”, λ = .321) had low standardized loadings, so they were dropped from the following analyses.

**Table F. CFA model estimates of the four-factor model.**

| **Factor** | **Item** | **Estimate** | **SE** | **z** |
| --- | --- | --- | --- | --- |
| Factor 1: Science-related activities | How often do you usually visit places related to science (e.g., observatories or botanical gardens) in your free time? | 1.00 |  |  |
|  | Art museums | 0.899 | 0.020 | 44.223 |
|  | Other museums (e.g., local or arts and crafts museums) | 0.868 | 0.020 | 43.814 |
|  | Science or technology museums | 1.057 | 0.022 | 47.869 |
|  | Science centres | 1.017 | 0.021 | 47.291 |
|  | Literature events | 0.677 | 0.021 | 32.341 |
|  | Planetariums | 0.779 | 0.020 | 38.826 |
|  | Lectures, talks or webinars related to science | 0.907 | 0.022 | 41.465 |
|  | National parks or other nature sites | 0.645 | 0.020 | 32.570 |
|  | Zoos or aquariums | 0.403 | 0.018 | 22.274 |
| Factor 2: Science self-efficacy | I don't think I'm smart enough to understand science. | 1.00 |  |  |
|  | I don't think I'm smart enough to understand technology. | 0.962 | 0.018 | 53.787 |
|  | I don't actually know what a scientist does. | 0.797 | 0.015 | 52.063 |
|  | I don't actually know what an engineer does. | 0.589 | 0.015 | 38.262 |
|  | Science is not for me. | 0.886 | 0.015 | 57.347 |
|  | I can well understand scientific terminology, such as hypothesis, theory, experiment and clinical trial. | -0.870 | 0.015 | -56.162 |
|  | I'm well informed about science, scientific research and their developments. | -0.931 | 0.015 | -60.938 |
|  | School put me off science. | 0.641 | 0.015 | 42.783 |
|  | I would feel comfortable in places where science is discussed and practiced, such as laboratories, science centres, and industrial environments. | -0.862 | 0.015 | -57.030 |
|  | Do you have relatives, friends or colleagues, who work with/in science? | -0.711 | 0.019 | -38.226 |
|  | Do you speak about things related to science with other people? How often? | -0.819 | 0.015 | -53.688 |
|  | I follow news about new technologies. | -0.774 | 0.015 | -50.423 |
| Factor 3: Early encouragement | My parents or guardians emphasised that science would be beneficial for me in the future. | 1.00 |  |  |
|  | My parents or guardians thought it was important for me to study science in school. | 0.973 | 0.020 | 48.506 |
|  | A significant adult (such as parent or teacher) encouraged me to continue science studies. | 0.935 | 0.019 | 49.229 |
|  | My parents or guardians were interested in science. | 0.878 | 0.018 | 48.887 |
|  | My teachers emphasised that science would be beneficial for me in the future. | 0.840 | 0.019 | 45.314 |
|  | My teachers thought I was gifted in science. | 0.852 | 0.018 | 46.604 |
| Factor 4: Science attitudes | Young people's interest in science is essential for our future welfare. | 1.00 |  |  |
|  | It's important for our society that young people understand science. | 1.156 | 0.031 | 37.078 |
|  | Science has so much significance in our lives that everyone should be interested in it. | 0.935 | 0.027 | 34.452 |
|  | Science is important for understanding the world. | 1.344 | 0.033 | 40.888 |
|  | Scientific knowledge is useful in my daily life. | 1.275 | 0.031 | 40.711 |
|  | The scientific knowledge I learned in school has been useful in my daily life. | 1.169 | 0.030 | 39.378 |
|  | The next generation has more opportunities for work due to science and technology. | 0.532 | 0.024 | 21.734 |
|  | I'm interested in scientific research and findings. | 1.472 | 0.034 | 43.134 |
|  | The mathematics I learned in school has been useful in my daily life. | 0.640 | 0.025 | 25.548 |
|  |  | **Covariances** | | |
| Factor 1 | Factor 2 | -.391 | .009 | -44.74 |
|  | Factor 3 | -.437 | .010 | -42.28 |
|  | Factor 4 | -.398 | .009 | -43.46 |
| Factor 2 | Factor 3 | .255 | .008 | 33.025 |
|  | Factor 4 | .290 | .009 | 32.936 |
| Factor 3 | Factor 4 | .287 | .009 | 33.39 |

*Note*. For all estimates *p* < .001.

**Table G. CFA model estimates of the four-factor model with a second-order factor representing science capital (Factor 5).**

| Factor | Item | Estimate | SE | z | p |
| --- | --- | --- | --- | --- | --- |
| Factor 1: Science-related activities | I visit places related to science (e.g., observatories or botanical gardens) in my free time. | 1.00 |  |  |  |
|  | Art museums | 0.900 | 0.020 | 44.246 | <.001 |
|  | Other museums (e.g., local or arts and crafts museums) | 0.870 | 0.020 | 43.836 | <.001 |
|  | Science or technology museums | 1.056 | 0.022 | 47.848 | <.001 |
|  | Science centres | 1.015 | 0.021 | 47.238 | <.001 |
|  | Literature events | 0.678 | 0.021 | 32.363 | <.001 |
|  | Planetariums | 0.777 | 0.020 | 38.747 | <.001 |
|  | Lectures, talks or webinars related to science | 0.906 | 0.022 | 41.437 | <.001 |
|  | National parks or other nature sites | 0.644 | 0.020 | 32.551 | <.001 |
|  | Zoos or aquariums | 0.401 | 0.018 | 22.142 | <.001 |
| Factor 2: Self-efficacy | I don't think I'm smart enough to understand science. | 1.00 |  |  |  |
|  | I don't think I'm smart enough to understand technology. | 0.962 | 0.018 | 53.796 | <.001 |
|  | I don't actually know what a scientist does. | 0.797 | 0.015 | 52.061 | <.001 |
|  | I don't actually know what an engineer does. | 0.589 | 0.015 | 38.252 | <.001 |
|  | Science is not for me. | 0.886 | 0.015 | 57.337 | <.001 |
|  | I can well understand scientific terminology, such as hypothesis, theory, experiment and clinical trial. | -0.871 | 0.016 | -56.170 | <.001 |
|  | I'm well informed about science, scientific research and their developments. | -0.931 | 0.015 | -60.943 | <.001 |
|  | School put me off science. | 0.641 | 0.015 | 42.800 | <.001 |
|  | I would feel comfortable in places where science is discussed and practiced, such as laboratories, science centres, and industrial environments. | -0.862 | 0.015 | -57.035 | <.001 |
|  | I have relatives, friends or colleagues, who work with/in science. | -0.711 | 0.019 | -38.237 | <.001 |
|  | I speak about things related to science with other people. | -0.819 | 0.015 | -53.672 | <.001 |
|  | I follow news about new technologies. | -0.773 | 0.015 | -50.411 | <.001 |
| Factor 3: Early encourage-ment | My parents or guardians emphasised that science would be beneficial for me in the future. | 1.00 |  |  |  |
|  | My parents or guardians thought it was important for me to study science in school. | 0.973 | 0.020 | 48.482 | <.001 |
|  | A significant adult (such as parent or teacher) encouraged me to continue science studies. | 0.934 | 0.019 | 49.188 | <.001 |
|  | My parents or guardians were interested in science. | 0.878 | 0.018 | 48.868 | <.001 |
|  | My teachers emphasised that science would be beneficial for me in the future. | 0.839 | 0.019 | 45.284 | <.001 |
|  | My teachers thought I was gifted in science. | 0.852 | 0.018 | 46.595 | <.001 |
| Factor 4: Science attitudes | Young people's interest in science is essential for our future welfare. | 1.00 |  |  |  |
|  | It's important for our society that young people understand science. | 1.158 | 0.031 | 37.091 | <.001 |
|  | Science has so much significance in our lives that everyone should be interested in it. | 0.936 | 0.027 | 34.458 | <.001 |
|  | Science is important for understanding the world. | 1.346 | 0.033 | 40.882 | <.001 |
|  | Scientific knowledge is useful in my daily life. | 1.277 | 0.031 | 40.699 | <.001 |
|  | The scientific knowledge I learned in school has been useful in my daily life. | 1.170 | 0.030 | 39.356 | <.001 |
|  | The next generation has more opportunities for work due to science and technology. | 0.531 | 0.024 | 21.724 | <.001 |
|  | I'm interested in scientific research and findings. | 1.475 | 0.034 | 43.121 | <.001 |
|  | The mathematics I learned in school has been useful in my daily life. | 0.638 | 0.025 | 25.473 | <.001 |
| Factor 5: Science capital | Factor 1 | 1.00 |  |  |  |
|  | Factor 2 | -1.599 | .038 | -42.18 | <.001 |
|  | Factor 3 | 1.062 | .026 | 40.43 | <.001 |
|  | Factor 4 | 1.103 | .031 | 36.09 | <.001 |

1. **Testing for metric invariance**

We fitted the model supported by the CFA results to the whole data and conducted measurement invariance tests for gender groups by first conducting the CFA separately for the two groups, and then restricting the factor loadings to be similar across the groups and comparing the model fits with the lavTestLRT() function. If the test proved significant, we examined which slopes were different between the gender groups with the univariate score test using the lavTestScore() function.

Only four respondents had indicated their gender as “other”, so they were left out from the measurement invariance tests. Some response categories for Factor 1 item “Planetariums” were zero in one group, and this item was dropped from the subsequent analyses. In Model 1, all factor loadings and covariances were freely estimated for the two gender groups. The model was a good fit to the data (Hu and Bentler, 1999): CFI = .984, TLI = .983, RMSEA = .063, 90%CI for RMSEA = [.061,.065], SRMR = .063), supporting configural invariance.

In Model 2, factor loadings were restricted to be equal in the two gender groups, and this model was compared to Model 1 to test for metric invariance. The model comparison showed that we failed to obtain metric invariance, χ^2^(30) = 265.59, p < .001. Univariate analyses of the factor loadings between the two groups showed that the loadings differed for several items in all four factors. As we failed to obtain metric invariance, gender was included as a grouping variable in the subsequent analyses.

1. **References**

Archer L, Dawson E, DeWitt J, Seakins A and Wong B (2015) “Science capital”: A conceptual, methodological, and empirical argument for extending bourdieusian notions of capital beyond the arts. Journal of Research in Science Teaching 52(7): 922–948.

Archer L and DeWitt J (2013) ASPIRES: young people’s science and career aspirations, age 10–14. Final project report, Department of Education and Professional Studies, King’s College London, UK.

Hu L and Bentler PM (1999) Cutoff criteria for fit indexes in covariance structure analysis: Conventional criteria versus new alternatives. Structural Equation Modeling: A Multidisciplinary Journal 6(1): 1–55.

Jorgensen TD, Pornprasertmanit S, Schoemann AM, Rosseel Y, Miller P, Quick C, Garnier-Villarreal M, Selig J, Boulton A, Preacher K, Coffman D, Rhemtulla M, Robitzsch A, Enders C, Arslan R, Clinton B, Panko P, Merkle E, Chesnut S, Byrnes J, Rights JD, Longo Y, Mansolf M, Ben-Shachar MS, Rönkkö M and Johnson AR (2022) semTools: Useful Tools for Structural Equation Modeling. R package version 0.5-6. Retrieved from: https://CRAN.R-project.org/package=semTools

Motta, M., Chapman, D., Haglin, K., & Kahan, D. (2021). Reducing the administrative demands of the science curiosity scale: a validation study. International Journal of Public Opinion Research, 33(2), 215-233.

Nassiri V, Lovik A, Molenberghs G, Verbeke G and Busch T (2021) mifa: Multiple Imputation for Exploratory Factor Analysis. R package version 0.2.0. Retrieved from: https://CRAN.R-project.org/package=mifa

R Core Team (2022) R: The R Project for Statistical Computing, Vienna, Austria. Available at: <https://www.R-project.org>

Revelle W (2023) psych: Procedures for Psychological, Psychometric, and Personality Research, Northwestern University, Evanston, Illinois. R package version 2.2.9. Available at: <https://CRAN.R-project.org/package=psych>

Rosseel Y (2012) lavaan: An R Package for Structural Equation Modeling. Journal of Statistical Software 48(2): 1–36.

UK Department for Business, Energy & Industrial Strategy (2019) Public attitudes to science 2019. BEIS Research Paper Number 2020/012, 17 July.

Van Buuren S and Groothuis-Oudshoorn K (2011) mice: Multivariate Imputation by Chained Equations in R. Journal of Statistical Software 45(3): 1–67.
